# Chicago College of Dental Surgery

**Published:** 1890-03

**Authors:** 


ARTICLE VII.

### CHICAGO COLLEGE OF DENTAL SURGERY.

---

The eighth annual commencement of the Chicago College of Dental Surgery, dental department of Lake Forest University, was held at the Chicago Opera House. Dr. Brophy read the annual report of the college, in which he showed its prosperous condition. From two graduates the first year they had grown until to-day they had a class of sixty. They had become a department in Lake Forest University and were proud of it. Then the degrees were conferred and the diplomas awarded by the faculty. William Lloyd Jones, D. D. S., was the class valedictorian. The orchestra played the class song, "Papa's Wee Girl," a new lullaby written and composed by F. A. Green, which was loudly applauded. The faculty address was delivered by Dr. A. W. Harlan. It was full of happy ideas. W. C. Roberts, D. D., LL. D., President of Lake Forest University, delivered the closing address of the afternoon.

The class of graduates is as follow:

Charles Edward Austin, B. S.; Frederick Douglas Axton, James Down Banes, Daniel Wesley Bottorf, John

Henry Chase, Joseph William Dostal, William Edward Emmons, L. D. S.; Harvey Everett Follansbee, Obe Edward Gibson, Linneaous Melbourn Goodearle, Earl Evlin Gould, Frank Albert Green, Edwin Grant Howard, Frank Sylvester Heer, Will Lloyd Jones, Richard Kempter, Halbert Eaton Kinney, Frank Kolar, George Wilson Toles, Ernest Lincoln Knapp, Frank Ambrose Lane, James Truman Lennington, Michael Leninger, Charles Beatty Magill, James Ralph Maguire, George Bruce Martin, Almon Green Moffett, James Doyle Moore, Joseph Gregory Pflaff, Guy M. Phelps, M. D.; George Edwin Zinn, B S.; John James Pountain, Harry Monroe Prickett, John Willett Putnam, Frederick Kent Ream, Edmund Walter Russell, Otto August Ruthenberg, Charles Carver Ryan, Grant John Roberts, Allan Benjamin Fernald, Fenwick Earl Salisbury, Frank Steece Schadel, James Adam Shoemaker, Albert Gustave Seeglitz, Jacob Hamlin Smyser, Melvin Wellington Swartz, Frederick Richard Suggitt, Lewis Solomon Tenny, Frederick Solomon Tinslar, Cornelius Nicholas Trompen, Rollin Brede Tuller, Orrin Thompson, James Lincoln Ubellar, John Quigley Waddell, Charles Herbert Waterhouse, M. D.; Charles Edward White, Herbert Cameron West, William Henry Conrad Wiesler, Charles Augustus Whitenack, Edward Everett Williams.

In the evening the alumni association gave their eighth annual dinner at the Laland Hotel. Dr. Brophy presided. President W. C. Roberts, D. D., LL. D., responded to the first toast, "The University and College," with a happy address that gave general satisfaction. Dr. Edward Noyes responded to the toast, "Dental Education of the Future." "The Graduate and His Relation to Dental Literature" was responded to by Dr. A. W. Harlan. "Dental Legislation, Beneficial and Detrimental," was responded to by Dr. C. R. E. Koch. "The World's Exposition and the Dentists of Chicago" was the subject to which Dr. Louis Ottofy responded. "The Profession Abroad" was responded to by Dr. J. A. Swasey. "The Alumni Association" fell to the lot of Dr. T. A. Broadbent. "The History of the Class of 1890" was the last toast and was responded to by Dr. L. S. Tenny. "The Class of '90", by Dr. F. D. Axton; "The Dental Protective Association," by Dr. J. N. Crouse; "Poem to the Class of '91," by Dr. F. A. Green; Presentation of cane to Dr, N. D. Edmonds by Dr. R. B. Tuller on behalf of the class of 1890.

---

### Communications.

### MISSOURI STATE DENTAL ASSOCIATION.

MEXICO, MO., March 15th, 1890.

*Editor of the American Journal of Dental Science*,

